# Monolithic dual-wedge prism-based spectroscopic single-molecule localization microscopy

**DOI:** 10.1515/nanoph-2021-0541

**Published:** 2022-01-21

**Authors:** Ki-Hee Song, Benjamin Brenner, Wei-Hong Yeo, Junghun Kweon, Zhen Cai, Yang Zhang, Youngseop Lee, Xusan Yang, Cheng Sun, Hao F. Zhang

**Affiliations:** Department of Biomedical Engineering, Northwestern University, 2145 Sheridan Rd., Evanston 60208, IL, USA; Department of Mechanical Engineering, Northwestern University, 2145 Sheridan Rd., Evanston 60208, IL, USA

**Keywords:** single-molecule localization microscopy, spectroscopy, super-resolution fluorescence imaging

## Abstract

By manipulating the spectral dispersion of detected photons, spectroscopic single-molecule localization microscopy (sSMLM) permits concurrent high-throughput single-molecular spectroscopic analysis and imaging. Despite its promising potential, using discrete optical components and managing the delicate balance between spectral dispersion and spatial localization compromise its performance, including nonuniform spectral dispersion, high transmission loss of grating, high optical alignment demands, and reduced precision. We designed a dual-wedge prism (DWP)-based monolithic imaging spectrometer to overcome these challenges. We optimized the DWP for spectrally dispersing focused beam without deviation and with minimal wavefront error. We integrated all components into a compact assembly, minimizing total transmission loss and significantly reducing optical alignment requirements. We show the feasibility of DWP using ray-tracing and numerical simulations. We validated our numerical simulations by experimentally imaging individual nanospheres and confirmed that DWP-sSMLM achieved much improved spatial and spectral precisions of grating-based sSMLM. We also demonstrated DWP-sSMLM in 3D multi-color imaging of cells.

## 1 Introduction

Spectroscopic single-molecule localization microscopy (sSMLM) simultaneously captures and accumulates spatial and spectral information of single-molecule fluorescence to simultaneously reconstruct super-resolution images of multiple molecular contrasts. It has become a powerful tool in cell biology and material science [1], [2], [3], [4], [5], [6], [7]. Compared with traditional single-molecule localization microscopy (SMLM) recording only the spatial information, sSMLM introduces additional dispersive optical components, such as prisms [1], [2], [3] or diffraction gratings [4], [5], [6], to collect the associated spectral signatures. The prism-based sSMLM system used a beam splitter and discrete optical components to divide the collected photons into two separate optical beam paths (one for spatial image and another for spectral image) [3]. The increased number of air/dielectric interfaces resulted in higher transmission loss. And the stringent requirements for aligning these discrete optical components further compromise the system reliability. Users need to have sophisticated optical alignment skills to achieve and maintain optimal performance, which imposes challenges to biological researchers.

In contrast, the grating-based sSMLM system unifies the photon-splitting and dispersion functions using a blazed diffraction grating. It separates the incident beam into the 0th and 1st diffraction orders, corresponding to the spatial and spectral images, respectively [5], [6], [7], [8], [9]. The reduced number of discrete components favorably improves system reliability and footprint. In addition, the linear dispersion of the grating brings an additional benefit to the ease of spectral analysis. However, the grating-based design has its limitations. Specifically, the large angular offset between the 0th and 1st diffraction orders makes the resulting spatial or spectral images vulnerable to geometrical and chromatic aberrations. Both aberrations may undermine the accuracy or precision of the reconstructed image and spectra analysis. Moreover, gratings generally have higher transmission losses (∼30%) than prisms, reducing photon budgets and imaging resolution [8].

This work reports a monolithic optical modular design that vastly reduces the number of discrete optical components for simplified optical configuration and alignment, significantly reduces transmission loss, and potentially improves system reliability. We designed a compact optical assembly using a dual-wedge prism (DWP), referred to as DWP spectrometer, to achieve 3D sSMLM with improved spatial precision.

## 2 DWP design

**Figure 1:**
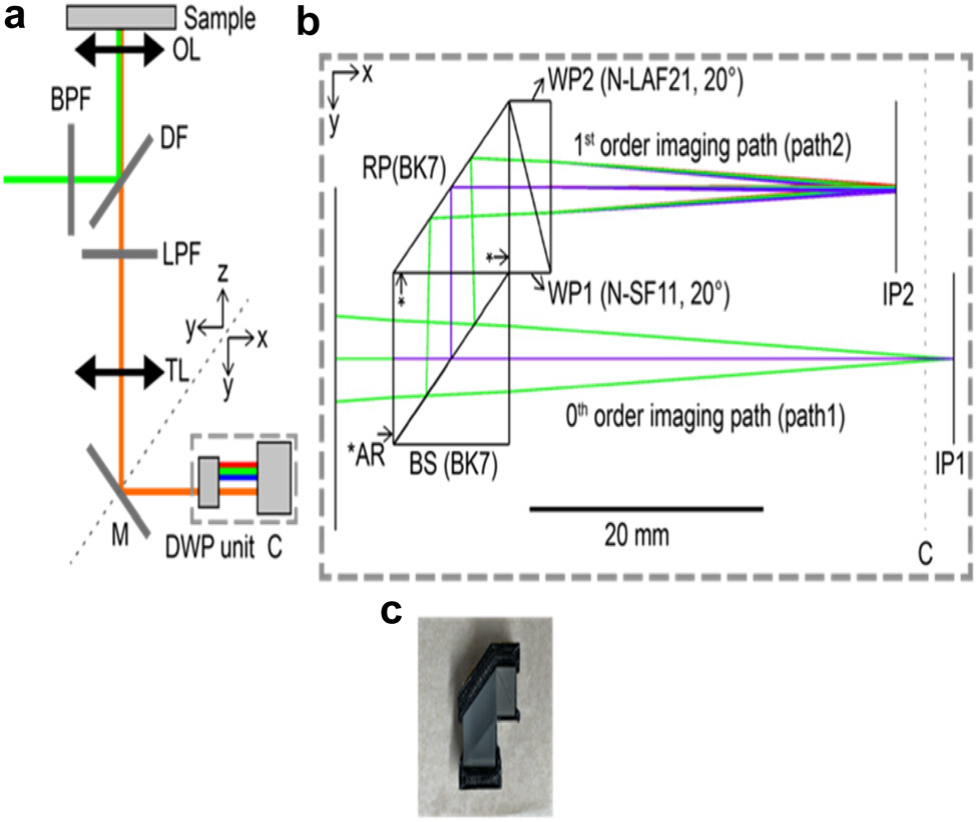
3D DWP sSMLM design. (a) Schematic of the 3D sSMLM system with the DWP unit; (b) Zemax design of the DWP unit optical assembly. (c) Picture of the DWP unit optical assembly, which consists of a customized DWP pair and a commercially available lateral beam splitter. BS: cube beam splitter; RP: right-angle prism; WP: wedge prism; AR: anti-reflection coating; OL: objective lens; BPF: band-pass filter; DF: dichroic filter; LPF: long-pass filter; TL: tube lens; M: mirror; DWP unit: dual-wedge prism unit; IP: image plane; C: camera plane.

Figure 1(a) shows the schematic of the 3D sSMLM system using the DWP unit. The overall system is based on an inverted optical microscope (Ti-U, Nikon) with an objective lens (OL, 100×, NA = 1.49, CFI apochromat TIRF lens, Nikon) and a matching tube lens (TL). We placed the DWP unit between the TL and a scientific complementary metal–oxide–semiconductor (sCMOS) camera (Prime95B, Photometrics). The filter set consists of a band-pass filter (BPF, FF01-642/10-25, Semrock), a dichroic mirror (FF649-DI01-25X36, Semrock), and a long-pass filter (LPF, BLP01-647R-25, Semrock) for fluorescent imaging. The fluorescence emission exiting the TL then passes the DWP unit to create spatial and spectral images.

Figure 1(b) illustrates the DWP spectrometer design, which consists of a cube beam splitter (BS, 50:50 split ratio), a right-angle prism (RP), and DWP for spectrally dispersing focused beam without deviation. The BS first splits the incident beam at a 50:50 split ratio. The transmitted beam (path 1) forms a spatial image on its image plane, IP1. The reflected beam (path 2) is further reflected by the RP along the *x*-axis and dispersed along the *y*-axis after passing through the DWP. The DWP unit consists of a pair of wedge prisms, which include one with strong chromatic dispersion (WP1, N-SF11 glass, refractive index *n* = 1.791 at 550 nm, Shanghai Optics) and another one with weak chromatic dispersion (WP2, N-LAF21 glass, refractive index *n* = 1.792 at 550 nm, Shanghai Optics) [10]. WP1 exhibiting strong chromatic dispersion is mainly used to disperse the incident beam to reveal the spectroscopic signatures. The resulting diversion angle of the refracted beam is subsequently compensated for by WP2 and, thus, the beam exiting WP2 remains parallel to the *x*-axis. It should be noted that WPs are known to introduce aberrations in the convergent light, resulting in imaging artifacts such as astigmatism [11]. Thus, we optimized the geometry and materials of WP1 and WP2 to minimize the wavefront error induced by DWP. Such design ensures the resulting spectral image plane (IP2) to be parallel with the spatial image plane (IP1) and minimizes the image aberration when we record both IP1 and IP2 using the same camera (C).

We adjusted the optical pathlength difference between path 1 and path 2 to ∼5 mm to create an axial separation between IP1 and IP2, enabling 3D biplane imaging [9]. We cemented all the optical components together to form the single DWP unit, as shown in Figure 1(b). The size of each subcomponent can be customized to fit specific experimental needs. As the spatial and spectral imaging paths are parallel with the *z*-axis, this monolithic imaging spectrometer can be inserted in-line into an existing imaging path of a commercial microscope. All exposed surfaces are anti-reflection coated to minimize transmission loss further. Figure 1(c) shows the picture of the DWP unit optical assembly, which consists of custom-made WPs and a commercially available lateral beam splitter (#47188, Edmund Optics).

## 3 DWP performance evaluation

**Figure 2:**
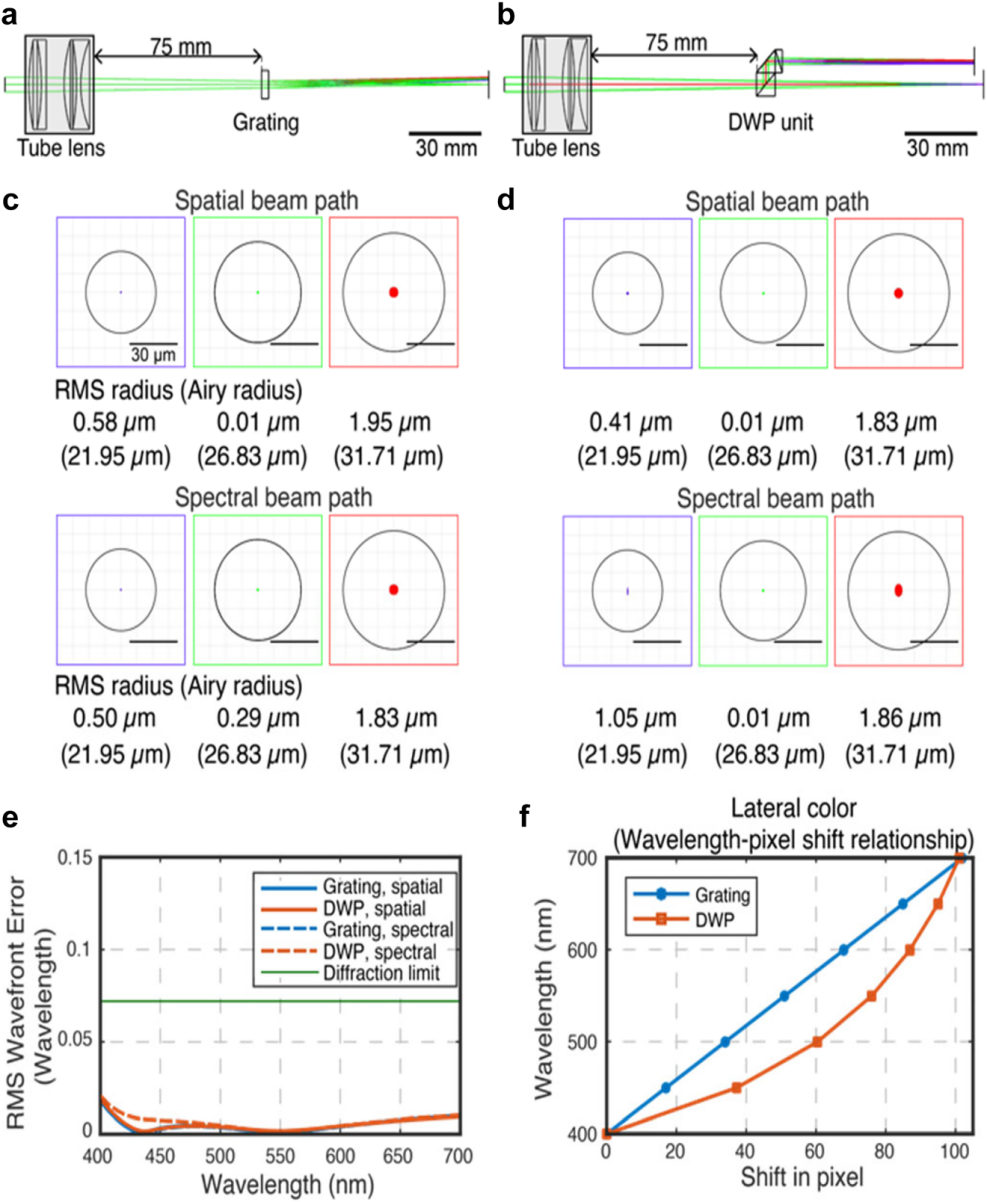
DWP performance evaluation using Zemax. Layouts of (a) the grating-based and (b) the DWP-based designs; simulated spot diagrams at different wavelengths (from left to right: 450, 550, and 650 nm; from the top to bottom: spatial and spectral beam paths) for (c) the grating-based and (d) the DWP-based designs; (e) simulated RMS wavefront errors of spatial and spectral beam paths in both designs; (f) wavelength-pixel shift relationship. We used a groove density of 50 grooves/mm, showing a comparable SD value to the DWP-based design using off-the-shelf optics. The overall SD of both designs is 3 nm/pixel. Dotted lines for the grating and DWP in (e) are mostly overlayed with solid lines. Scale bar: 30 µm in (c) and (d).

We compared the performances of grating-based and DWP-based sSMLM using Zemax^®^. We show their optical layouts in Figure 2(a) and (b), respectively. In both designs, we focused the incident beam using a TL modeled as a microscope component as previously reported [12]. We placed both the grating and the DWP unit 75-mm away from the TL.

Figure 2(c) and (d) show the simulated spot diagrams of the spatial and spectral beam paths in both designs at three representative wavelengths (450 nm, 550 nm, and 650 nm). Overall, the root-mean-square (RMS) radiuses in both designs are significantly lower than the reference diffraction-limited criterion, indicating that both designs offer diffraction-limited performances. For example, we observed a 0.58-µm RMS radius in the grating-based design and a 0.41-µm RMS radius in the DWP-based design, both of which are significantly smaller than the 21.95-µm Airy disk radius at 450 nm. Also, the simulated RMS wavefront errors of spatial and spectral beam paths are compared with the diffraction-limited case, as shown in Figure 2(e). The wavefront errors in both designs are significantly lower than the reference diffraction-limited criterion (0.08*λ*) across the entire simulated range. These results suggest that our DWP design effectively minimized the wavefront errors. We further investigated the performance of DWP-based and grating-based sSMLM for off-axis incident beams. We first assumed a 3.5-mm offset with respect to an optical axis in front of a dispersive element along the *x*- and *y*-axes, which shows the performance at different camera sensor positions after passing through the dispersive element. Also, we assumed a 7-mm offset in front of a tube lens along the *x*- and *y*-axes. As shown in Figures S1 and S2, we observed that the RMS radiuses in both cases are significantly less than the reference diffraction-limited criterion, indicating that both designs offer diffraction-limited performances even for the off-axis incident beam.

We experimentally measured the wavefront error of the DWP module using a Shack–Hartmann Wavefront Sensor (WFS40-5C, Thorlabs). As shown in Figure S3, we plot the coefficients of the Zernike polynomials corresponding to the two orders of the DWP module with the first few terms representing pseudo-aberrations of piston, tip, and tilt omitted. Using the coefficients and Eq. (S1), we obtained values of 0.011 and 0.015 for the 0th and 1st orders of the DWP, respectively, showing a good agreement with our Zemax simulation. Details of the experimental procedure are provided in the Supplementary Material.

Figure 2(f) compares the simulated wavelength–pixel shift relationship, which quantifies the achievable spectral dispersion (SD), in the grating-based and DWP-based design with a camera pixel size of 11 µm [13]. The grating-based design is known for its linear dispersion (the blue curve). In contrast, the DWP-based design shows a nonlinear dispersion (the orange curve), which can be fitted using a third-order polynomial equation [1]. Overall, both designs provide an SD of ∼6.5 nm/pixel. The SD can be tuned either by changing the wedge angles of the DWP or translating the DWP module along the optical axis, as labeled in Figure 2(b), in a converging beam path [6].

We theoretically compared the spatial and spectral precisions of the DWP-based and the grating-based designs [6, 8]. According to the manufacturer’s specifications, we assumed an absolute transmission loss (at 680 nm) of 30% by the grating in the grating-based sSMLM system, and 5% by the BS, 0.5% by the RP, 2% by the DWP in the DWP-based sSMLM system. Considering a given quantum efficiency of the selected sCMOS camera (85% at 680 nm) and an experimentally measured ∼2:3 split ratio between the spatial and spectral channels, the absolute transmission efficiencies of the spatial and spectral images in the grating-based sSMLM system are 24 and 36% at 680 nm, respectively. The absolute transmission efficiencies of the spatial and spectral images in the DWP-based sSMLM system are 40 and 39%, respectively. Then, we estimated the lateral spatial precision Δx using [14]

(1) $$\Delta x^{2}=\frac{\sigma^{2}+a^{2}/12}{N}\left(1+4\tau +\sqrt{\frac{2\tau }{1+4\tau }}\right)\text{,}$$

where $\tau =2\pi \left(b+n_{\text{ro}}^{2}\right)\left(\sigma^{2}+a^{2}/12\right)/Na^{2}$ ; σ [nm] is the standard deviation of the Gaussian function; a [nm] is the back-projected pixel size; N is the number of detected photons; b is the number of background photons per pixel; and $n_{\text{ro}}$ [e−] is the readout noise. We estimated the spectral precision $\sigma_{\lambda }$ using [13]

(2) $$\sigma_{\lambda }^{2}=\frac{s_{\lambda }^{2}}{N}+\frac{1024n_{\text{bg}}^{2}s_{\lambda }^{3}s_{x}}{3\Delta \lambda \Delta xN^{2}}+\frac{1024n_{\text{ro}}^{2}s_{\lambda }^{3}s_{x}}{3\Delta \lambda \Delta xN^{2}}\text{,}$$

where $s_{\lambda }$ [nm] and $s_{x}$ [nm] are the standard deviations of the Gaussian function along the spectral-axis and the *x*-axis, respectively; $n_{\text{bg}}^{2}$ is the number of background photons per pixel; Δλ is the SD [nm/pixel]; Δx [nm] is the back-projected pixel size along the *x*-axis.

**Figure 3:**
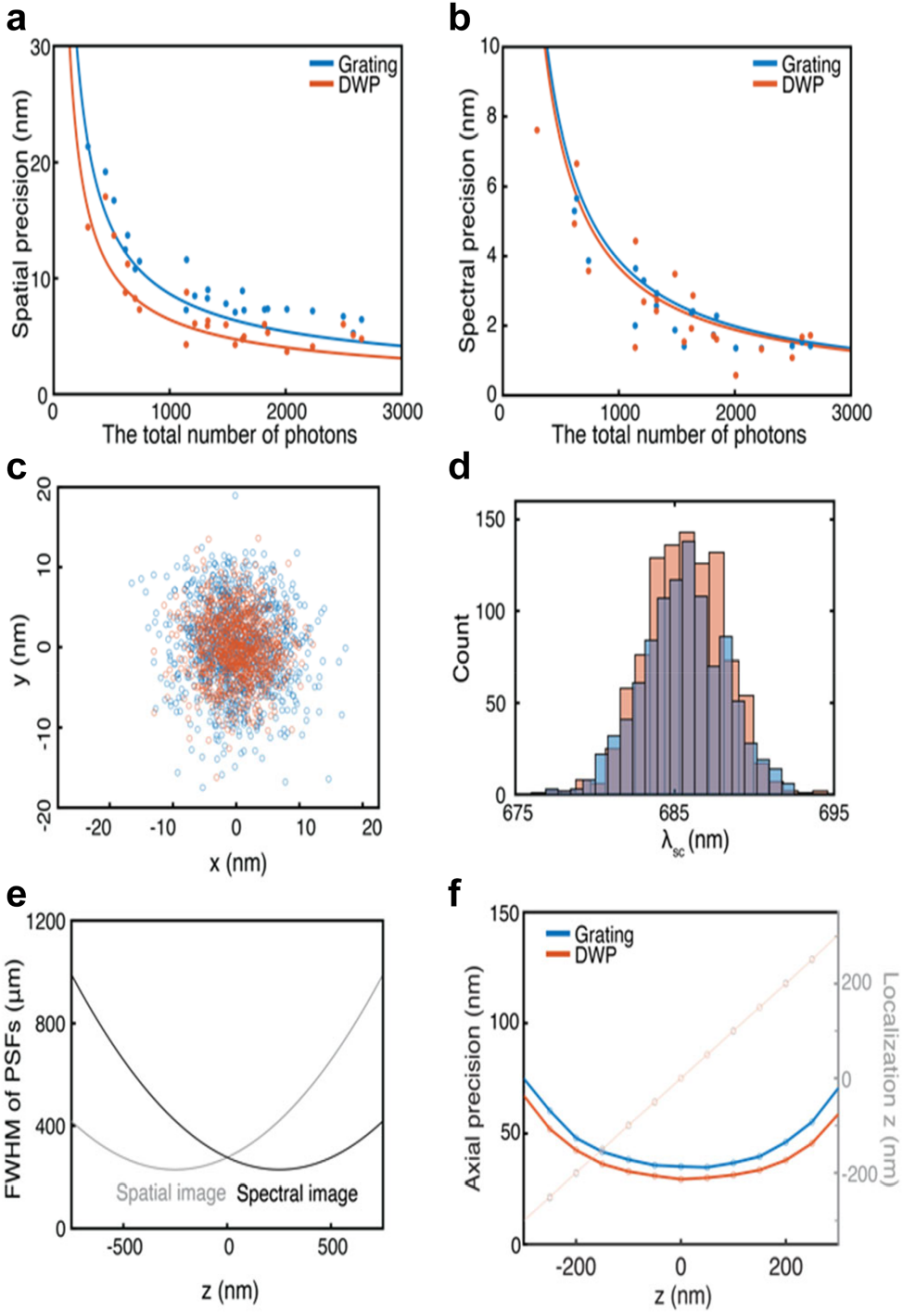
DWP performance evaluation using numerical and analytical simulations. (a) Theoretical and experimental lateral spatial precision and (b) spectral precision of the grating-based (the blue color) and the DWP-based (the orange color) designs. The lines and dots indicate the theoretical and experimental values, respectively. (c) Scatterplot of spatial precision and (d) histogram of spectral precision of both designs at 2000 total photons. (e) Depth calibration curve of 3D biplane imaging. (f) Theoretically estimated axial spatial precision of the grating-based and the DWP-based designs.

As shown in Figure 3(a), we observed that the DWP-based design offers an improved lateral spatial precision of 4.2 nm, representing a 25% improvement over the grating-based design’s spatial precision (5.6 nm) when the total number of photons is 2000. The higher transmission efficiency of the dispersive prism than the grating contributes primarily to such precision improvement. Meanwhile, these two designs provide comparable spectral precisions (1.9 nm for the DWP-based design and 2.0 nm for the grating-based design) due to their similar absolute transmission efficiencies and spectral dispersion in the spectral channel, as shown in Figure 3(b).

We performed a numerical simulation to estimate the spatial and spectral precisions and compared them with the results obtained by the analytical model. We used the previously reported numerical simulation method [7, 13, 14] and provided all the key parameters in Table 1. From the numerical simulation, we found that the spatial precisions in the DWP- and grating-based designs were 4.8 and 6.6 nm, respectively, as shown in Figure 3(c), when the total photons were 2000. We observed comparable spectral precisions in the DWP- and grating-based designs as 2.5 and 2.6 nm, respectively (Figure 3(d)), closely matching the analytical solution. These results suggest that the DWP-based design reduced the system complexity and improved photon utilization efficiency.

**Table 1:** List of parameters used in analytical solutions.

For Δ*x*	*σ* (nm)	*a* (nm)	*b* (photons/pixel)	*n*_ro_ (e−)		
	97	110	20	1.6		
For *σ*_*λ*_	*s*_*λ*_ (nm)	*s*_*x*_ (nm)	$n_{\text{bg}}^{2}$ (photons/pixel)	*n*_ro_ (e−)	Δ*λ* (nm/pixel)	Δ*x* (nm)
	17	97	20	1.6	6.5	110

We further extended the numerical simulation to 3D imaging to estimate the axial spatial precision of the DWP-based design. As shown in Figure 2(b), we achieved an optical path difference of ∼5 mm between the spatial (IP1) and spectral (IP2) image planes, which generates an axial separation of ∼500 n/m using a 100X OL [15]. We first generated an ideal 3D calibration curve for biplane imaging based on this axial separation, as shown in Figure 3(e) [9]. The calibration curves show the full-width-at-half-maximum (FWHM) values of the PSFs along the *x*-axis in both spatial and spectral images. We used these calibration curves to generate PSFs at different depths as the ground truth. We used these ground-truth PSFs to estimate the axial precision of the biplane sSMLM system in the same manner as we previously reported [9]. Other key parameters used in this simulation are listed in Table 2. Figure 3(f) shows the simulated axial precision (the left vertical axis) and the reconstructed localization z-position (the right vertical axis). For example, we obtained the axial precision of 29.3 nm for the DWP-based design and 35.0 nm for the grating-based design, respectively, at a 0-nm depth, 2000 photons, and a background of 20 photons/pixel, corresponding to a 16% improvement.

**Table 2:** List of parameters used in simulations.

Camera	Pixel size (µm)	Quantum efficiency @ 680 nm (%)	Readout noise (e−)	Signal (photons)	Background (photons/pixel)	Standard deviation of PSF (nm)	Iteration number	Precision	Spectral dispersion (nm/pixel)	Axial separation (nm)
Prime 95B	11	85	1.6	2000	20	97	10,000	For lateral	–	–
								For spectral	6.5	–
								For axial	–	500

In addition, we experimentally validated the simulated results using far-red nanospheres (F8807, Thermo Fisher) as shown in Figure 3(a) and (b). For this validation, we immobilized the nanospheres on glass substrates and measured the spatial and spectral precisions matching the simulation conditions. We adjusted the laser input power from ∼0.2 to 0.5 W cm^−2^ to generate different photon budgets of individual nanospheres. We first measured the baseline of the nanobeads without a dispersive element to determine the total photon budget of each nanosphere with an exposure time of 10 ms. Next, we performed two separate measurements to estimate the spatial and spectral precisions under each corresponding illumination condition. The spatial precision is estimated using the standard deviation of the average *x* and *y* coordinates of a single nanosphere localized using ThunderSTORM [16] over 50 consecutive frames. The spectral precision is estimated using the standard deviation of the spectral centroid (SC) over 50 consecutive frames, where the SC is the intensity-weighted mean along the wavelength axis [13]. We applied a drift correction algorithm to the localizations by subtracting the values with a trendline smoothed with a Savitzky–Golay filter [17]. We observed that the experimental results matched well with the theoretical models.

Using the validated simulation models, we further estimated the precision improvements of the DWP design compared with the grating-based design at a different splitting ratio, as shown in Figure S4. Compared with grating-based sSMLM at 1:3 splitting ratio [8, 9] offered at 570 nm, DWP improves lateral spatial precision by 47% (from 8.1 to 4.3 nm) and maintains a similar spectral precision (2.6 nm for the DWP-based design and 2.2 nm for the grating-based design) at a photon budget of 2000. It also improves the axial precision by 23% (from 36.4 to 28.2 nm) in bi-plane 3D imaging.

## 4 Imaging validation

### 4.1 Sample preparation

We experimentally demonstrated multi-color 3D imaging of U2OS cells using the DWP-based system. We labeled microtubules and mitochondria using AF647 and CF680, respectively. We maintained U2OS cells in Dulbecco’s modified Eagle medium (DMEM, Gibco/Life Technologies) supplemented with 2-mM L-glutamine (Gibco/Life Technologies), 10% fetal bovine serum (Gibco/Life Technologies), and 1% penicillin and streptomycin (100 U/mL, Gibco/Life Technologies) at 37 °C. We fixed the cells with 3% paraformaldehyde and 0.1% glutaraldehyde in phosphate-buffered saline (PBS) for 10 min and rinsed them with PBS twice. Then, we permeabilized the cells with a buffer (3% bovine serum albumin (BSA) and 0.5% Triton X-100 in PBS for 10 min), blocked in 2.5% goat serum in PBS for 30 min, and incubated with the primary antibodies in the blocking buffer (2.5% goat serum in PBS) overnight in the refrigerator with rotation. For primary antibody incubation, we used mouse anti-TOM20(F-10) (1:100 dilution. 200 μg/mL, sc-17764, Santa Cruz) and rabbit anti-B-tubulin (1:100 dilution, 200 μg/mL, PAS-16863, Thermo Fisher Scientific). We washed the samples with washing buffer (0.2% BSA and 0.1% Triton X-100 in PBS) for 5 min three times and incubated with secondary antibodies labeled with mouse CF680 (1:100 dilution, 100 μg/mL, donkey anti-mouse IgG-CF680) for TOM20 and rabbit AF647 (1:100 dilution, 100 µg/mL, donkey anti-rabbit IgG-AF647) for B-Tubulin for 30 min. Then, we washed the cells with washing buffer (0.2% BSA and 0.1% Triton X-100 in PBS) twice and with PBS twice for 5 min each and stored them in PBS at 4 °C. We used an imaging buffer (pH = ∼8.0, 50 mM Tris, 10 mM NaCl, 0.5 mg mL^−1^ glucose oxidase (G2133, Sigma-Aldrich), 2000 U/mL catalase (C30, Sigma-Aldrich), 10% (w/v) D-glucose, and 100 mM cysteamine) for imaging.

### 4.2 sSMLM imaging

**Figure 4:**
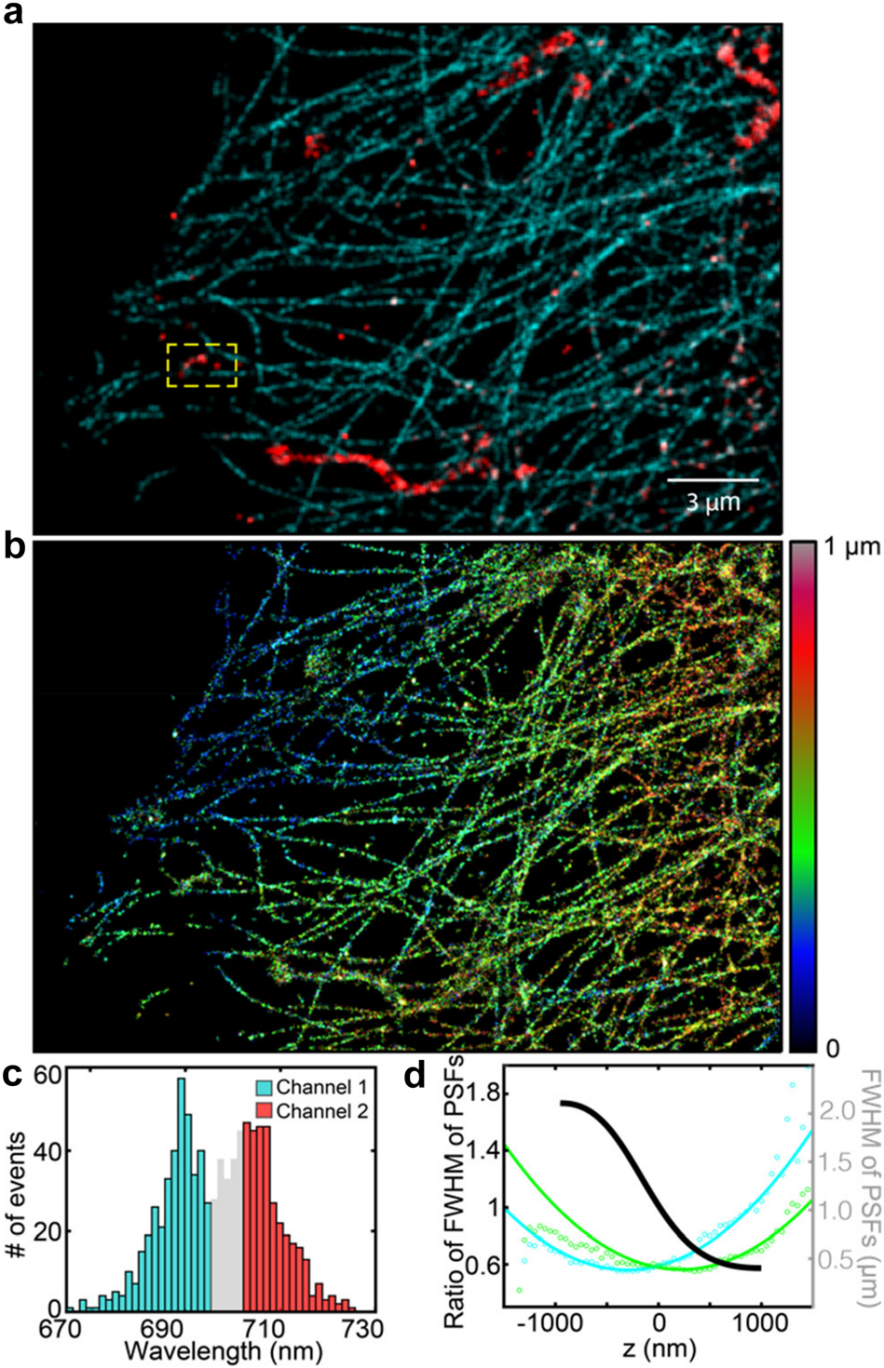
Demonstration of 3D multi-color DWP imaging. (a) Two-color 2D and (b) 3D sSMLM reconstruction image of microtubules (cyan) and mitochondria (red) labeled with AF647 and CF680. 3D reconstruction image is visualized with pseudo colors corresponding to the axial coordinates of individual molecules. (c) The SC distributions of AF647 and CF680 signals enclosed by the yellow dashed box in panel (a). (d) Experimentally obtained 3D bi-plane calibration curve. Different contrasts were applied to (a) and (b) for better visualization in the reconstruction.

Figure 4(a) and (b), respectively, shows the two-color 2D and 3D sSMLM reconstruction images. We used a weighted SC method to identify individual molecules in multi-color imaging [5, 7, 9, 13]. We first calculated SCs of individual molecules and classified them by defining different spectral windows: the first window, the cyan color in Figure 4(a), is below 697 nm, and the second window, the red color in Figure 4(a), is above 705 nm. Figure 4(c) shows the SC distributions of AF647 and CF680 within the yellow dashed box highlighted in Figure 4(a). We reconstructed 3D images using the biplane method as previously reported [9]. We first estimated the full-width-at-half-maximum (FWHM) values of the PSFs along the *x*-axis in both spatial and spectral images. Then, we assigned axial coordinates of individual localizations based on the 3D calibration, as shown in Figure 4(d). Lastly, we visualized 3D reconstruction image with pseudo colors corresponding to the axial coordinates of each molecule.

**Figure 5:**
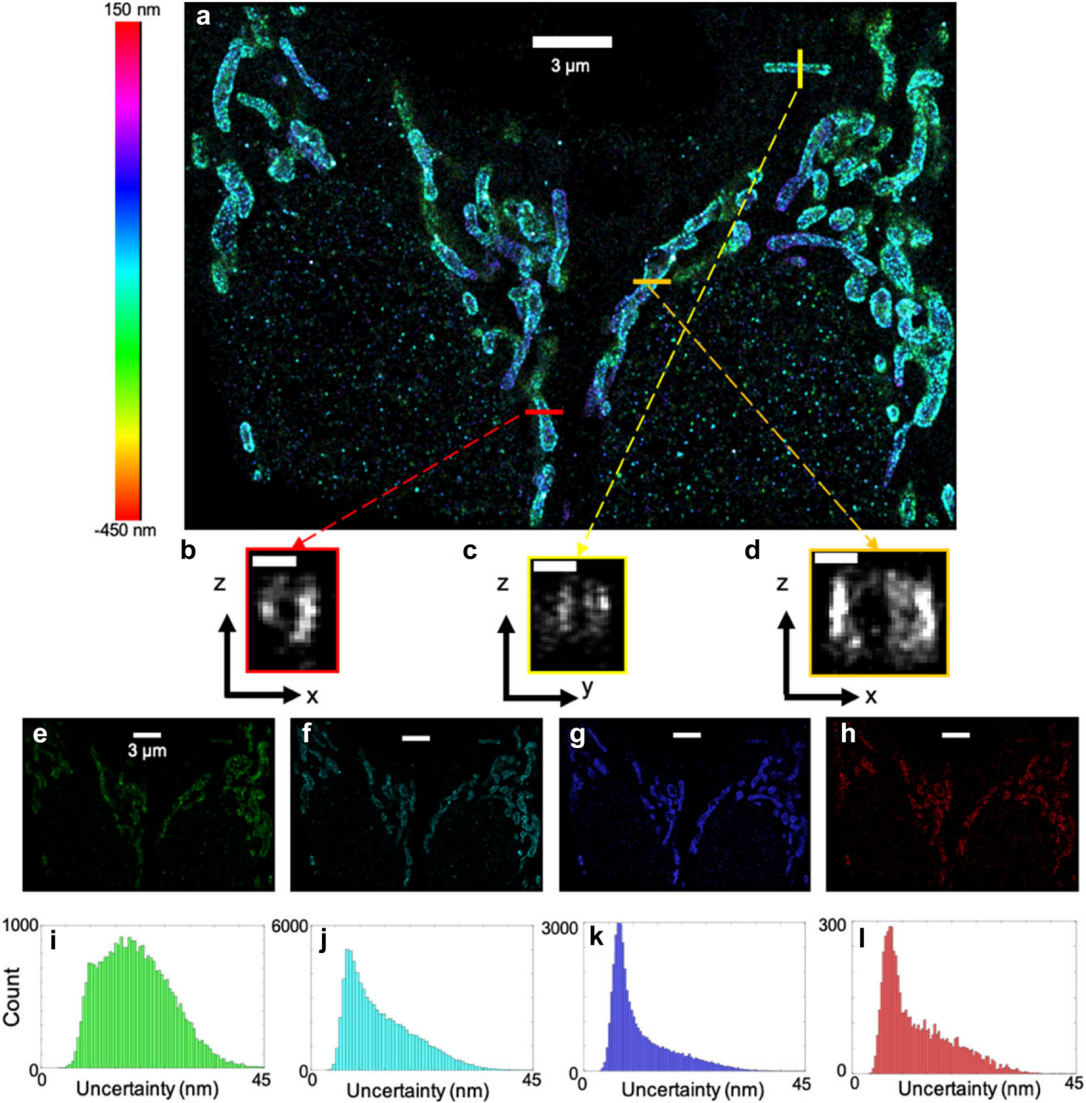
Demonstration of 3D single-color DWP imaging. (a) 2D projection of a 3D mitochondria image color-coded with respect to *z*-position. The pseudocolor spectrum ranges from magenta to red and represents a 600 nm range. (b–d) Cross-sections of the mitochondria from the three positions in the panel (a) highlighted by the red, yellow, and orange lines, respectively. The scale bar is 300 nm (e–h) 2D projections of four 50-nm thick slices of mitochondria and (i–l) the corresponding histograms of the lateral localization uncertainties estimated using Thunderstorm. Slices correspond to −255:−205 nm (e, i), −135:−85 nm (f, j), −35:15 nm (g, k), and 65–115 nm (h, l), respectively. The size of inset boxes in (a) are exaggerated for better highlighting.

To characterize the 3D imaging using the DWP, we performed a single-color 3D imaging using HeLa cell mitochondria labeled with AF647. Figure 5(a) shows a 2D projection image with pseudocolors corresponding to the axial position of each molecule. By visualizing orthogonal cross-sections across the mitochondria as shown in Figure 5(b)–(d), we successfully confirmed the 3D ring structure associated with the outer-mitochondrial membrane.

In addition, we visualized the *x*–*y* cross-sections and the histograms of the lateral localization uncertainty at different heights, as shown in Figure 5(e)–(l). We measured the localization uncertainty at different *z*-slices of 50 nm width ranging from −225 to 115 nm. We estimated the lateral uncertainty for each localization using Eq. (1) and plotted a histogram for each slice. As expected, the axial slice where the spatial image was in focus corresponded to the lowest lateral uncertainty, as illustrated in Figure 5(i)–(l), while the slices in which the spatial image was out of focus corresponded to higher spatial uncertainties.

To further test the imaging resolution of the 2D projection in Figure 5(a), we performed Fourier Ring Correlation (FRC) [18], which is a method employed used in SMLM to determine overall resolution, incorporating both precision and sparsity of estimated localizations. As illustrated in Figure S5, we obtained an imaging resolution of 49.4 nm at a threshold value of 1/7, which indicates the maximum spatial frequency that holds structural information.

### 4.3 Spectral calibration

We imaged a nanohole array using a white-light lamp and 550 (FB550-10, Thorlabs), 605 (#86356, Edmund optics), 642 (FF01-642/10-25, Semrock), 685 (#86738, Edmund Optics), and 750 nm (FB750-10, Thorlabs) BPFs. The nanohole array contains 5 holes with a spacing of 2 µm along the *x*-axis and 5 µm along the *y*-axis. This calibration image included multiple PSFs of nanoholes in a spatial image and corresponding spectral PSFs in a spectral image. By integrating the PSFs in the spectral image along the *y*-axis, we obtained the emission peaks defined by the BPFs. Then, we obtained a calibration curve by fitting the wavelengths of the emission peaks with their corresponding pixel distances using a third-order polynomial function. For multi-color imaging, we fitted the wavelengths of the emission peaks using a first-order polynomial function because the pixel–wavelength relationship within the 650–750 nm is considered linear. Its pixel–wavelength variation within the range is less than 1 pixel. Details of PSF shapes and color identification procedure are provided in Figures S6 and S7 in the Supplementary Material.

## 5 Conclusions

In this work, we designed and demonstrated a compact 3D sSMLM module with the monolithic imaging spectrometer that can readily transform a conventional optical microscope into an sSMLM. Using ray-tracing and numerical simulations, we estimated theoretically and experimentally achievable spatial and spectral precisions. We also compared the results with those acquired by an existing grating-based design with different splitting ratios. Finally, we validated its spectroscopic 3D multi-color imaging capability in cells, which shows the potential to uncover rich information about single molecules for the fundamental understanding of subcellular structures and nanomaterial properties. The reported 3D sSMLM module eliminates the need for time-consuming alignment and maintenance of discrete optical components by manufacturing a single unit of the compound optical element. We anticipate that the monolithic nature of the DWP unit allows for easy integration with commercial fluorescence microscope bodies and makes sSMLM broadly accessible by users in the biology research community.

## Supplementary Material

Supplementary Material

## References

[1] Zhang Z., Kenny S. J., Hauser M., Li W., Xu K. (2015). Ultrahigh-throughput single-molecule spectroscopy and spectrally resolved super resolution microscopy. *Nat. Methods*.

[2] Mlodzianoski M. J., Curthoys N. M., Gunewardene M. S., Carter S., Hess S. T. (2016). Super-resolution imaging of molecular emission spectra and single molecule spectral fluctuations. *PLoS One*.

[3] Comtet J., Glushkov E., Navikas V. (2019). Wide-field spectral super-resolution mapping of optically active defects in hexagonal boron nitride. *Nano Lett.*.

[4] Dong B., Almassalha L., Urban B. E. (2016). Super-resolution spectroscopic microscopy via photon localization. *Nat. Commun.*.

[5] Bongiovanni M. N., Godet J., Horrocks M. H. (2016). Multi-dimensional super-resolution imaging enables surface hydrophobicity mapping. *Nat. Commun.*.

[6] Zhang Y., Song K.-H., Dong B. (2019). Multi-color super-resolution imaging using spectroscopic single-molecule localization microscopy with optimal spectral dispersion. *Appl. Opt.*.

[7] Song K.-H., Zhang Y., Brenner B., Sun C., Zhang H. F. (2020). Symmetrically dispersed spectroscopic single-molecule localization microscopy. *Light Sci. Appl.*.

[8] Davis J. L., Zhang Y., Yi S. (2020). Super-resolution imaging of self-assembled nanocarriers using quantitative spectroscopic analysis for cluster extraction. *Langmuir*.

[9] Song K.-H., Zhang Y., Wang G., Sun C., Zhang H. F. (2019). Three-dimensional biplane spectroscopic single-molecule localization microscopy. *Optica*.

[10] Suzuki Y., Tani T., Sutoh K., Kamimura S. (2002). Imaging of fluorescence spectrum of a single fluorescent molecule by prism-based spectroscopy. *FEBS Lett.*.

[11] Howard J. W. (1985). Formulas for the coma and astigmatism of wedge prisms used in converging light. *Appl. Opt.*.

[12] Kurvits J. A., Jiang M., Zia R. (2015). Comparative analysis of imaging configurations and objectives for Fourier microscopy. *J. Opt. Soc. Am. A*.

[13] Song K.-H., Dong B., Sun C., Zhang H. F. (2018). Theoretical analysis of spectral precision in spectroscopic single-molecule localization microscopy. *Rev. Sci. Instrum.*.

[14] Rieger B., Stallinga S. (2014). The lateral and axial localization uncertainty in super-resolution light microscopy. *ChemPhysChem*.

[15] Prabhat P., Ram S., Ward E. S., Ober R. J. (2004). Simultaneous imaging of different focal planes in fluorescence microscopy for the study of cellular dynamics in three dimensions. *IEEE Trans. NanoBioscience*.

[16] Ovesný M., Křížek P., Borkovec J., Švindrych Z., Hagen G. M. (2014). ThunderSTORM a comprehensive ImageJ plug-in for PALM and STORM data analysis and super-resolution imaging. *Bioinformatics*.

[17] Savitzky A., Golay M. J. E. (1964). Smoothing and differentiation of data by simplified least squares procedures. *Anal. Chem.*.

[18] Banterle N., Bui K. H., Lemke E. A., Beck M. (2013). Fourier ring correlation as a resolution criterion for super-resolution microscopy. *J. Struct. Biol.*.

