# Supplementary material for: Monolithic dual-wedge prism-based spectroscopic single-molecule localization microscopy
Source: Nanophotonics. 2022 Jan 21;11(8):1527–35. doi: 10.1515/nanoph-2021-0541 (PMC9307059; doi:10.1515/nanoph-2021-0541)
Supplement: Supplementary file 1 — Supplementary Material [file j_nanoph-2021-0541_suppl.docx]

Ki-Hee Song, Benjamin Brenner, Wei-Hong Yeo, Junghun Kweon, Zhen Cai, Yang Zhang, Youngseop Lee, Xusan Yang, Cheng Sun and Hao F. Zhang*

Monolithic dual-wedge prism-based spectroscopic single-molecule localization microscopy: Supplementary Material

Abstract: This document provides supplementary information to “Monolithic dual-wedge prism-based spectroscopic single-molecule localization microscopy”.

1. Performance comparisons of grating-based and DWP-based sSMLM using Zemax®.


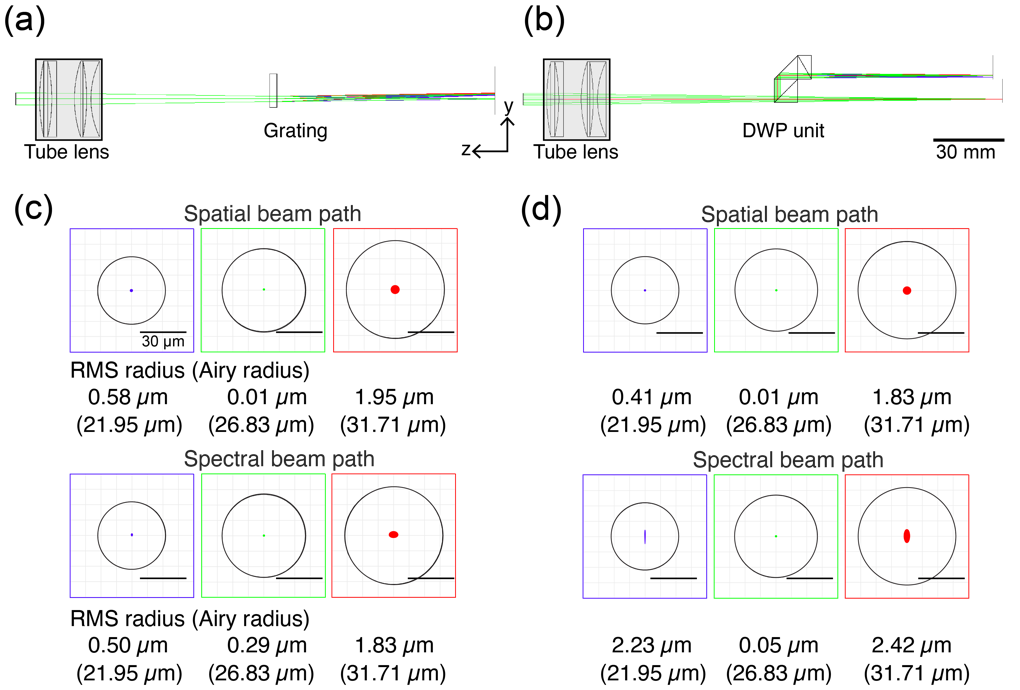


**Fig. S1:** Zemax simulation layouts of (a) the grating-based and (b) the DWP-based designs for a 3.5-mm offset with respect to an optical axis in front of a dispersive element along the x- and y- axes; Simulated spot diagrams at different wavelengths (from left to right: 450, 550, and 650 nm; Scale bar: 30 µm in (c) and (d).


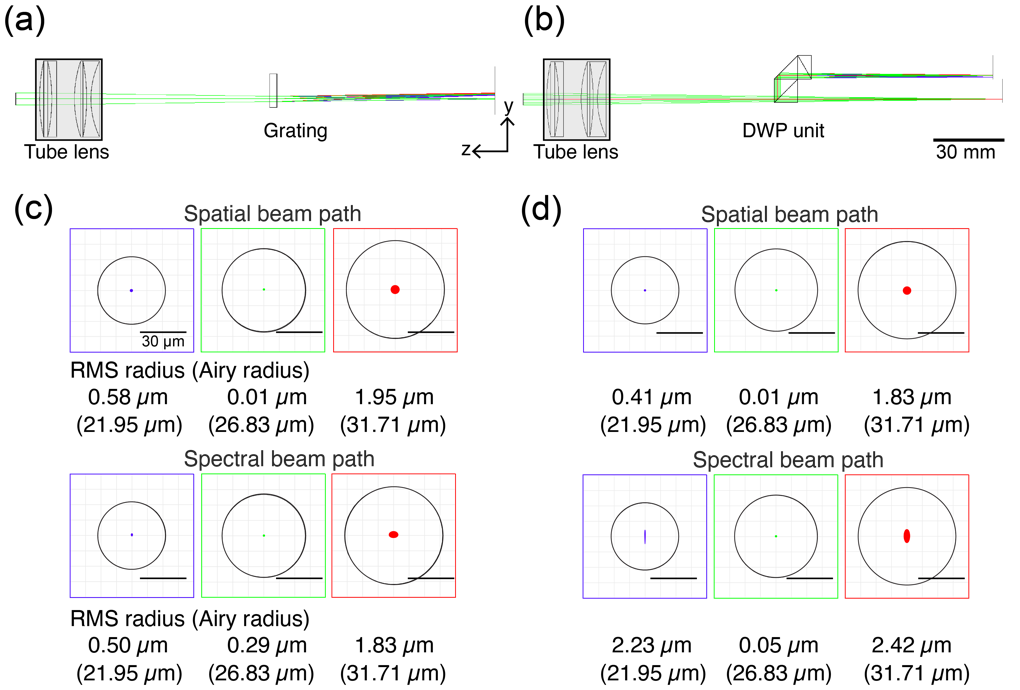


**Fig. S2:** Zemax simulation layouts of (a) the grating-based and (b) the DWP-based designs for a 7.0-mm offset with respect to an optical axis in front of a tube lens along the x- and y- axes; Simulated spot diagrams at different wavelengths (from left to right: 450, 550, and 650 nm; Scale bar: 30 µm in (c) and (d).

1. Wavefront error measurement of DWP

We experimentally measured the wavefront error of the DWP module. We used a Shack-Hartmann Wavefront Sensor (SHWS, WFS40-5C, Thorlabs) with an optical setup described as shown in Fig. S3(a). We used a HeNe laser (632.8 nm) with its beam expanded by ~4 times and collimated, then used a 200-mm lens to replicate a focusing beam entering the DWP module to match the tube lens present in the Nikon microscope used in our setup. The beam was then recollimated using a 150 mm lens before measuring it with the SHWS.

To measure the wavefront error of the DWP module, we first calibrated the sensor without the DWP module and then measured the 0^th^ and 1^st^ order images with the DWP module inserted. In Fig. S3(b), we plot the coefficients of the Zernike polynomials corresponding to the two orders of the DWP module with the first few terms representing pseudo-aberrations of piston, tip, and tilt omitted.


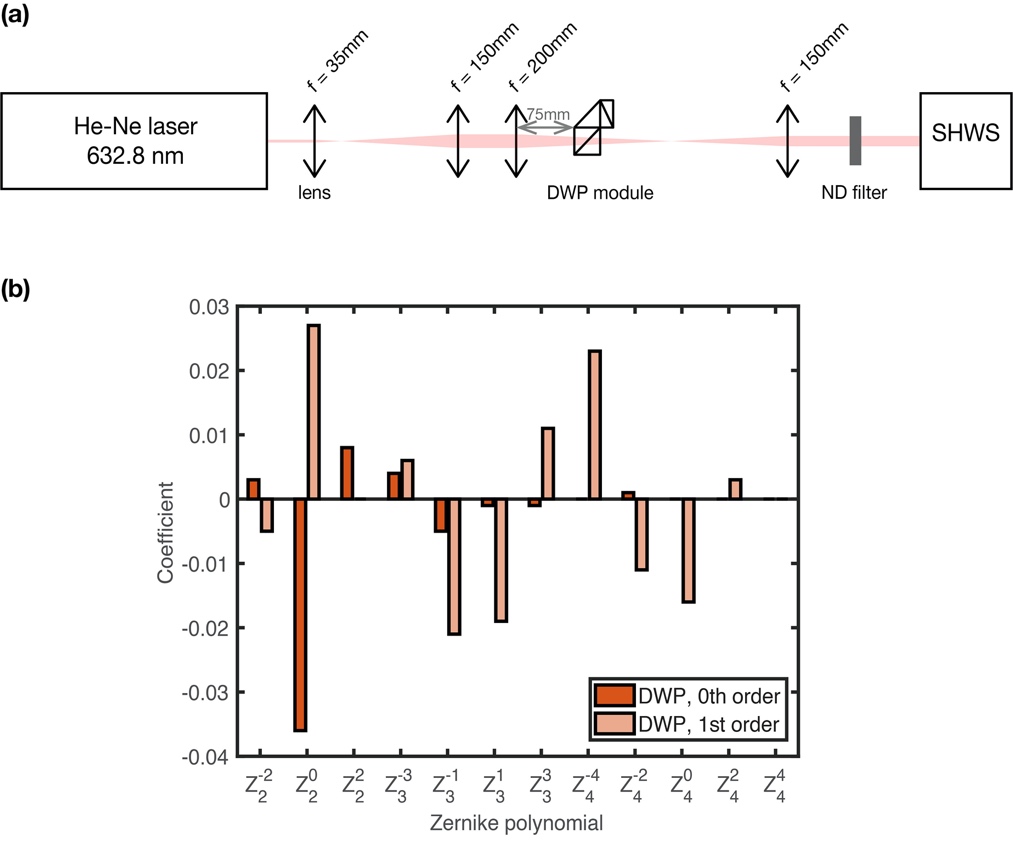


**Fig. S3:** (a) Schematic of the experimental setup to measure the wavefront using an SHWS. (b) Experimentally measured Zernike coefficients of the DWP module

We computed the RMS wavefront errors using the following formula [1]:

$\sigma=\left( \sum_{j=2} \left( C_{j}^{k} \right)^{2} \right)^{\frac{1}{2}}$, (S1)

where $C_{j}^{k}$ is the coefficient of each Zernike polynomial $Z_{j}^{k}$. We obtained values of 0.011 and 0.015 for the 0^th^ and 1^st^ orders of the DWP, respectively, which agreed well with our Zemax simulation results shown in Fig. 2.

1. Comparison of theoretical precisions the grating-based- and the DWP-based designs at a different splitting ratio


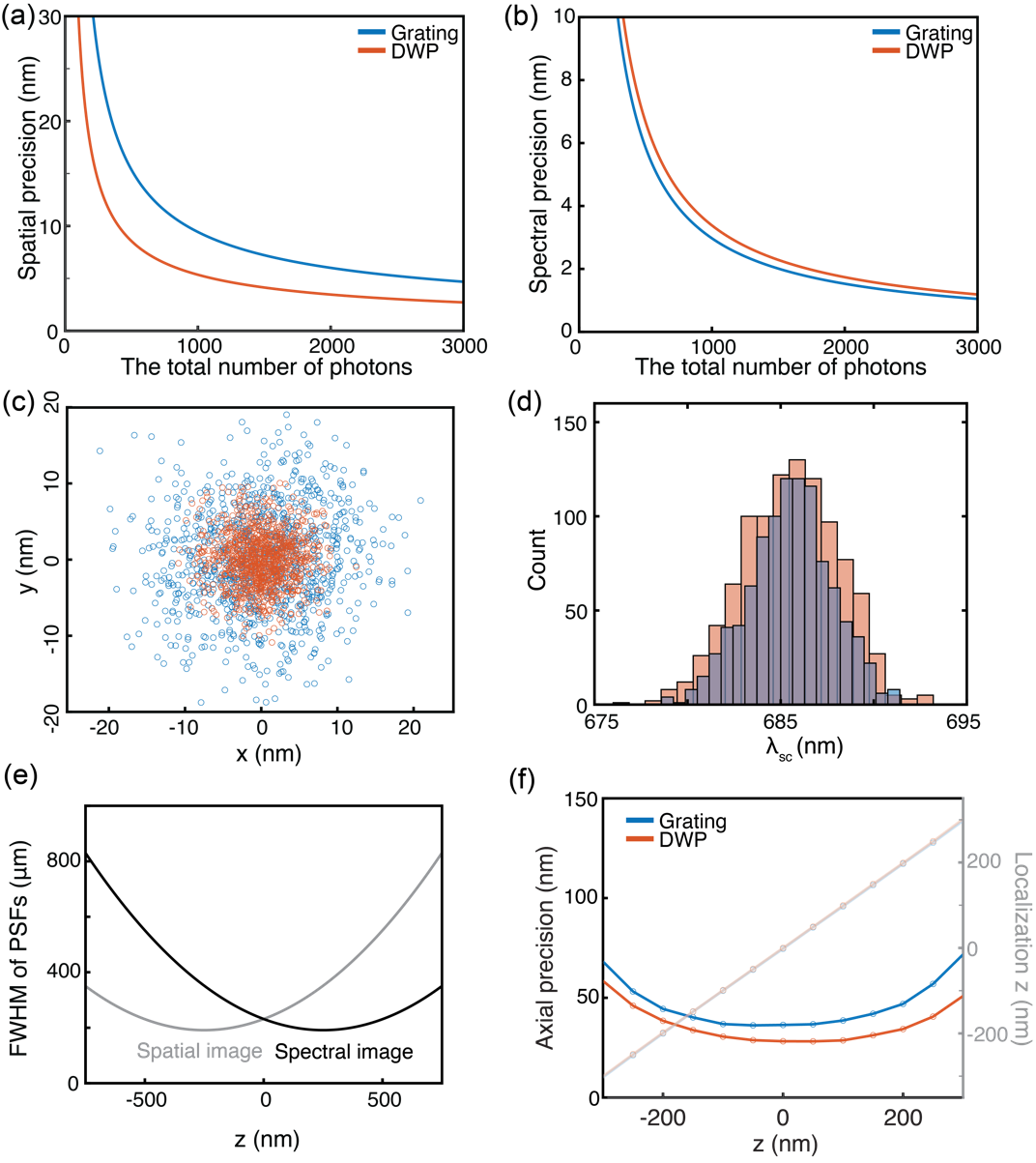


Fig. S**4:** (a) Theoretical lateral spatial precision and (b) spectral precision of the grating-based (the blue color) and the DWP-based (the orange color) designs. (c) Scatterplot of spatial precision and (d) histogram of spectral precision of both designs at 2000 total photons. (e) Depth calibration curve of 3D biplane imaging. (f) Theoretically estimated axial spatial precision of the grating-based and the DWP-based designs.

1. FRC evaluation


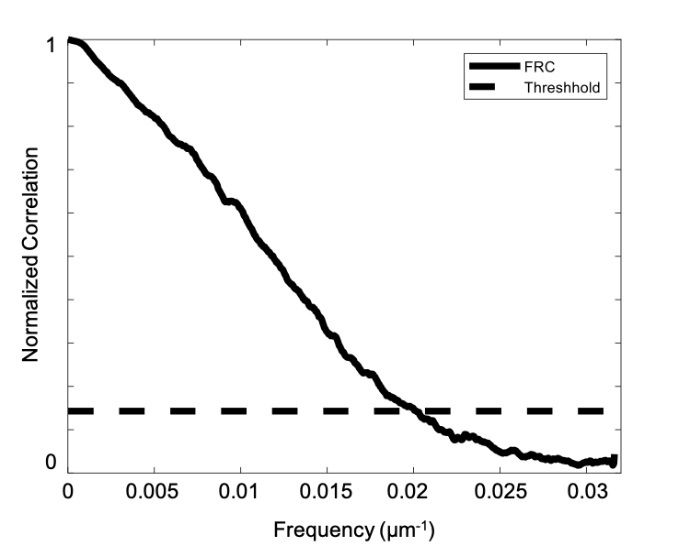


Fig. S5: FRC of the 2D projection image from Fig. 5(a). The solid black line represents the FRC, while the dashed line represents the resolution threshold.

1. PSF for 3D imaging and color identification

The Fig. S6(a) shows spatial PSFs as a function of depth used for generating 3D calibration curve while the Fig. S6(b) shows corresponding spectral PSFs. Specifically, we acquired a stack of images of a 200-nm red nanosphere, and set the objective lens focus to shift in intervals of 20 nm through a range of 3 µm. We visualized the XZ and YZ projections corresponding to the spatial image (Fig. S6(a)) and spectral images (Fig. S6(b)), respectively. The spectral PSF is asymmetrical in the XY direction as it only contains spectral information in the x-direction. And, it has a different axial focal point than the zeroth-order as it experiences a longer path length.


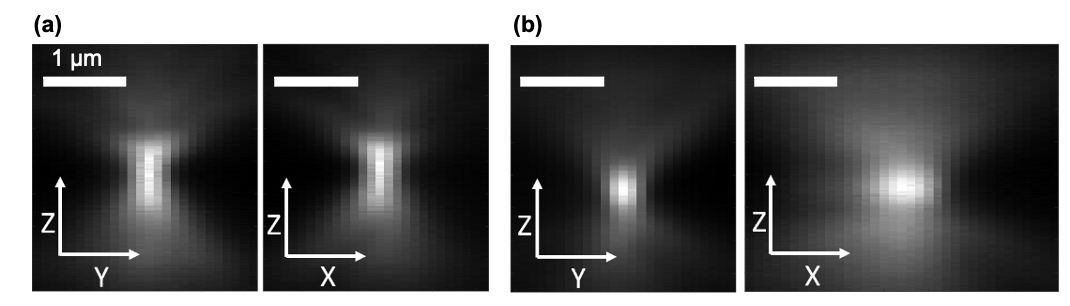


Fig. S6: (a) Spatial PSFs as a function of depth used for generating 3D calibration curve. (b) Corresponding spectral PSFs


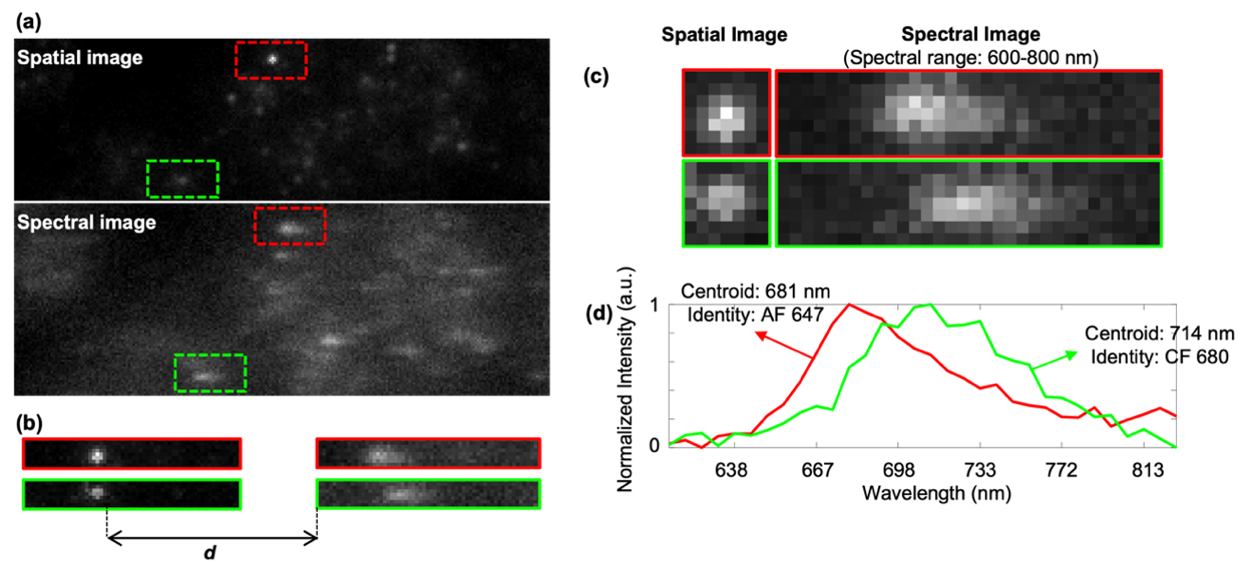


Fig. S7: Color identification of the DWP imaging (a) Single frame of DWP cell imaging data. Spatial image (top) and spectral image (bottom). The red box indicates AF647, which labels phosphorylated RNA polymerase II and the green box represents CF680, which labels the histone mark H3K27ac. (b) Magnified view of the PSFs marked with the colored box in (a). (c) Aligned PSFs with the same spatial and spectral pixel windows. (d) Emission spectra of the PSFs.

In order to identify the color information of individual molecules, we measured spectral centroids (SCs) after the spectral calibration procedure. Fig. S7 describes the procedure of estimating the SC. Specifically, Fig. S7(a) shows a single frame of DWP cell imaging data. The red box indicates AF647 labeling phosphorylated RNA polymerase II, and the green box represents CF680 labeling histone mark H3K27ac (Fig. S7(b)). First, we aligned individual PSFs with the same spatial and spectral pixel windows, using a known distance *d* between spatial and spectral images (Figs. S7(b)&(c)). Then, we summed the spectral PSFs in the y-direction and normalized them to create a 1D outline of each spectrum (Fig. S7(d)). The green line shows the spectrum of CF680, while the red line shows the spectrum of AF647. Finally, we calculated the SCs. Based on the SC values of individual molecules, we identify the molecules and characterize their color.

Reference

[1] V. Lakshminarayanan and A. Fleck, “Zernike polynomials: a guide”, Journal of Modern Optics **58**, 545-561,
(2011).
